# Supplementary material for: Biomarker Panel for the Diagnosis of Pancreatic Ductal Adenocarcinoma
Source: Cancers (Basel). 2020 Jun 1;12(6):1443. doi: 10.3390/cancers12061443 (PMC7352313; doi:10.3390/cancers12061443)
Supplement: Supplementary file 1 [file cancers-12-01443-s001.pdf]

Supplemental ***Materials***

Biomarker Panel for the Diagnosis of Pancreatic Ductal Adenocarcinoma

Hongbeom Kim, Kyung Nam Kang, Yong Sung Shin, Yoonhyeong Byun, Youngmin Han, Wooil Kwon, Chul Woo Kim and Jin-Young Jang

Table S1. Result of comparing 2 to 5 markers combinations with 6 marker panels among markers.

Marker	Training and Test Set				Validation Set			
	AUC	Accuracy (%)	Specificity (%)	Sensitivity (%)	AUC	Accuracy (%)	Specificity (%)	Sensitivity (%)
Panel vs Combination of 2 markers (RF)								
Panel	0.992	95	95	96	0.993	95	96	93
Max. CA19-9 + TTR	0.989	95	95	94	0.993	96	96	97
Min. B2M + CEA	0.686	76	95	18	0.8	81	97	30
Panel vs Combination of 3 markers (RF)								
Panel	0.992	95	95	96	0.993	95	96	93
Max. CA19-9 + TTR + D-Dimer	0.991	95	95	95	0.991	96	95	98
Min. CA125 + B2M + CEA	0.726	79	95	29	0.815	80	93	40
Panel vs Combination of 4 markers (RF)								
Panel	0.992	95	95	96	0.993	95	96	93
Max. CA19-9 + TTR + CRP + ApoA2	0.993	95	95	94	0.997	96	96	97
Min. CA125 + B2M + CEA + LRG1	0.774	80	95	33	0.841	81	95	37
Panel vs Combination of 4 markers (GLM)								
Panel	0.983	94	95	92	0.983	94	95	92
CA19-9 + TTR + CRP + ApoA2	0.982	95	95	94	0.986	95	96	93
Panel vs Combination of 4 markers (GLM + RF)								
Panel	0.984	94	95	92	0.986	95	96	92
CA19-9 + TTR + CRP + ApoA2	0.986	95	95	93	0.995	97	97	95
Panel vs Combination of 4 markers (RIDGE)								
Panel	0.987	95	95	93	0.985	95	96	92
CA19-9 + TTR + CRP + ApoA2	0.986	95	95	94	0.986	95	96	93
Panel vs Combination of 4 markers (SVM)								
Panel	0.990	95	95	95	0.991	97	98	92
CA19-9 + TTR + CRP + ApoA2	0.989	95	95	94	0.992	97	97	95
Panel vs Combination of 5 markers (RF)								

Panel	0.992	95	95	96	0.993	95	96	93
Max. CA19-9 + TTR + CEA + ApoA2 + CRP	0.993	95	95	95	0.997	96	95	97
Min. CA125 + B2M + CEA + LRG1 + CYFRA21.1	0.845	82	95	41	0.873	94	98	40
Panel vs Combination of 5 markers (GLM)								
Panel	0.983	94	95	92	0.983	94	95	92
CA19-9 + TTR + CEA + ApoA2 +CRP	0.981	95	95	94	0.987	95	95	93
Panel vs Combination of 5 markers (GLM + RF)								
Panel	0.984	94	95	92	0.986	95	96	92
CA19-9 + TTR + CEA + ApoA2 +CRP	0.984	95	95	94	0.997	97	97	97
Panel vs Combination of 5 markers (RIDGE)								
Panel	0.987	95	95	93	0.985	95	96	92
CA19-9 + TTR + CEA + ApoA2 +CRP	0.986	95	95	93	0.987	95	96	93
Panel vs Combination of 5 markers (SVM)								
Panel	0.990	95	95	95	0.991	97	98	92
CA19-9 + TTR + CEA + ApoA2 +CRP	0.988	95	95	95	0.992	96	98	92

AUC, area under the curve; RF, random forest; CA19-9, carbohydrate antigen 19-9; TTR, transthyretin; CEA, carcinoembryonic antigen; B2M, beta-2 microglobulin; CA125, cancer antigen 125; ApoA2, Apolipoprotein A2; CRP, C-reactive protein; LRG1, leucine rich alpha-2-glycoprotein 1; GLM, generalized linear model; SVM, support vector machine; CYRFA12.1, cytokeratin 19 fragment 21.1.

© 2020 by the authors. Licensee MDPI, Basel, Switzerland. This article is an open access article distributed under the terms and conditions of the Creative Commons Attribution (CC BY) license (http://creativecommons.org/licenses/by/4.0/).
